# Development of an objectively measured walkability index for the Netherlands

**DOI:** 10.1186/s12966-022-01270-8

**Published:** 2022-05-02

**Authors:** Thao Minh Lam, Zhiyong Wang, Ilonca Vaartjes, Derek Karssenberg, Dick Ettema, Marco Helbich, Erik J. Timmermans, Lawrence D. Frank, Nicolette R. den Braver, Alfred J. Wagtendonk, Joline W. J. Beulens, Jeroen Lakerveld

**Affiliations:** 1 Amsterdam UMC, Vrije Universiteit Amsterdam, Department of Epidemiology and Data Science, Amsterdam Public Health research institute, Boelelaan 1089a, 1081HV, Amsterdam, Netherlands; 2 Department of Human Geography and Spatial Planning, Faculty of Geosciences, Utrecht University, Princetonlaan 8a, 3584 Utrecht, CB Netherlands; 3 Julius Center for Health Sciences and Primary Care, University Medical Center Utrecht, Utrecht University, Utrecht, Netherlands; 4 Global Geo Health Data Center, University Medical Center Utrecht & Utrecht University, Utrecht, Netherlands; 5 Upstream Team, Vrije Universiteit, Amsterdam, Netherlands; 6 Department of Physical Geography, Utrecht University, Princetonlaan 8a, 3584 Utrecht, CB Netherlands; 7 Department of Urban Studies and Planning, UC San Diego, La Jolla, San Diego, USA; 8 Urban Design 4 Health, Inc, Rochester, NY USA

**Keywords:** Walkability, Validation, Physical activity, Built environment, Transport

## Abstract

**Background:**

Walkability indices have been developed and linked to behavioural and health outcomes elsewhere in the world, but not comprehensively for Europe. We aimed to 1) develop a theory-based and evidence-informed Dutch walkability index, 2) examine its cross-sectional associations with total and purpose-specific walking behaviours of adults across socioeconomic (SES) and urbanisation strata, 3) explore which walkability components drive these associations.

**Methods:**

Components of the index included: population density, retail and service density, land use mix, street connectivity, green space, sidewalk density and public transport density. Each of the seven components was calculated for three Euclidean buffers: 150 m, 500 m and 1000 m around every 6-digit postal code location and for every administrative neighbourhood in GIS. Componential z-scores were averaged, and final indices normalized between 0 and 100. Data on self-reported demographic characteristics and walking behaviours of 16,055 adult respondents (aged 18–65) were extracted from the Dutch National Travel Survey 2017. Using Tobit regression modelling adjusted for individual- and household-level confounders, we assessed the associations between walkability and minutes walking in total, for non-discretionary and discretionary purposes. By assessing the attenuation in associations between partial indices and walking outcomes, we identified which of the seven components drive these associations. We also tested for effect modification by urbanization degree, SES, age and sex.

**Results:**

In fully adjusted models, a 10% increase in walkability was associated with a maximum increase of 8.5 min of total walking per day (95%CI: 7.1–9.9). This association was consistent across buffer sizes and purposes of walking. Public transport density was driving the index’s association with walking outcomes. Stratified results showed that associations with minutes of non-discretionary walking were stronger in rural compared to very urban areas, in neighbourhoods with low SES compared to high SES, and in middle-aged (36–49 years) compared to young (18–35 years old) and older adults (50–65 years old).

**Conclusions:**

The walkability index was cross-sectionally associated with Dutch adult’s walking behaviours, indicating its validity for further use in research.

**Supplementary Information:**

The online version contains supplementary material available at 10.1186/s12966-022-01270-8.

## Background

Increased walking is associated with a range of favourable health outcomes, including reductions in cardiovascular risk and all-cause mortality [1, 2]. Globally, more than a quarter of the adult population does not meet the recommended level of at least 150 min of moderate-intensity physical activity (PA) per week [3]. In the Netherlands, non-adherence is even higher- at more than 50% [4]. Promotion of walking is therefore a promising public health intervention.

Socio-ecological models suggest that PA behaviours such as walking are influenced by a range of factors at both the individual as well as environmental levels [5–8]. Following this conceptual model, research has not only focused on personal motivations for PA but also on the social and environmental determinants of PA, due to the latter’s potential in guiding population-wide approaches to increase overall PA [9].

Properties of the built environment; in particular land use mix, population density, street connectivity and commercial floor area ratio; have been identified as correlates of walking [10, 11]. While studies mostly examine these components individually, a composite approach has been increasingly deployed in order to capture their co-occurrence; to reduce multicollinearity, over adjustment and measurement error; and to create actionable indices for policy applications [11–14]. Moreover, a composite walkability index could potentially account for more variations in walking compared to individual components alone [12]. Walkability indices have been widely constructed and studied, with systematic reviews indicating large consistency across studies in associations with walking [15, 16]. WalkScore® (Redfin, Seattle, WA USA), a commercially available walkability index, was associated with increased walking in Cuba [17], Canada [18] and with increased moderate to vigorous PA in the United States of America [19].

Despite its extensive application in environmental epidemiological research in North America and Australia [20–22], walkability indices have had limited application in Europe and especially the Netherlands. Dutch studies on walkability so far have focused on individual components [23, 24], specific sub-populations such as older adults [25], children [26, 27], patients of type-2 diabetes [28] or only zoomed in on a local geographical region [29].

A national perspective on walkability is still lacking, despite the potential of such an index in explaining differences in physical and socioeconomic contexts between countries [30]. The selection of components to include in this novel index should be based on both conceptual framework of walkability and the evidence derived from Dutch studies. The former include common walkability components guided by transport theories such as the 6D’s framework developed by Ewing and Cervero (2010) [31]. Beyond these common components, the incorporation of local evidence is important to nuance walkability measures, as exemplified by Herrmann et al. (2017) in their efforts to apply WalkScore in local context of Montreal, Canada [32]. They found that the addition of parking lots, setbacks and on-street canopies improved the predictive power of WalkScore.

Albeit still limited, Dutch walkability studies suggest that a walkability index should capture the geographical contrast in walkability between urban and rural areas, in mobility patterns between weekdays and weekends, between different purposes (e.g., walking for transport versus walking for leisure) [23], and between different age groups [33]. Notably, capturing these contrasts prove challenging in any context even outside the Netherlands [32]. We therefore aimed to compose a theory-driven walkability index for the Netherlands and to explore the associations between this index and walking behaviours of Dutch adults. Specifically, we examined how these associations differed between discretionary and non-discretionary purposes of walking and the effect modification of urbanization degrees, neighbourhood socioeconomic status (SES), age and sex. In addition, we investigated which walkability component was driving the index’s associations with walking outcomes.

## Methods

### Study population

Data from the Dutch National Travel Survey (OViN) for 2017 were used to estimate walking behaviours for a representative sample of the Dutch population. Details of this survey were described extensively elsewhere [34]. Briefly, the survey asked respondents to report their mobility for a pre-determined date to ensure all days of the year were sampled. Mobility information included starting points, destinations, purpose, means of transport and travel duration for every trip taken on that specified date. To prevent selection bias, respondents were selected based on both demographic characteristics and geographic distribution. Moreover, a mixed surveying method was employed: respondents were first invited to fill out a web-based form, or answer through the phone, or if non-response persists, were visited by an experienced surveyor personally. Since the invitation included the survey date, recall bias could be minimized. In addition, relevant personal and household data (e.g., gender, age, ethnic background, and household composition) were collected in the questionnaire. All walkability indices were coupled to respondents’ residential addresses on the basis of their 6-digit postal code, a relatively fine-grained geographical area consisting of about 20 home addresses on average.

### Walkability index composition

#### Selection of components

We employed a theory-driven, evidence-informed approach in selecting components for the walkability index. Built upon the transport literature, the 6D’s framework (including density, diversity, design, demand management, destination accessibility and distance to transit) could be applied in the case of built environment and walking [31] (Table 1). Specifically for walkability, the basic components such as street connectivity (intersection density), population density, retail area floor density, and land-use mix have been derived from foundational studies on walkability [12]. On top of that, we considered incorporating built environmental features positively associated with walking such as sidewalk density, green space, public transport availability, pedestrian safety, and street aesthetics from recent systematic reviews [10, 35–40]. Despite the limited literature on walkability in the Netherlands, some evidence for blue space [24, 41] and distance to food outlets [23, 41] was found, prompting us to also consider them for inclusion in the walkability index. After screening above-mentioned variables for data availability in terms of spatial and temporal coverages (national coverage, close to the year 2016) as well as spatial resolution, we ended up with seven components in the final index: population density, street connectivity, retail and service density, land use mix, green space, sidewalk density and public transport density (Table 1). For detailed description and technical GIS operationalisation of this walkability index, we refer to Wagtendonk and Lakerveld [42].

**Table 1:** The 6D’s framework with example and adaptations to the current study [31]

D	Examples	Included in current study
Density	Household/ Population density Job density Commercial floor area ratio	Population density Retail and service density
Diversity	Land use mix Job housing balance Distance to amenities	Land use mix (including food)
Design	Intersection density Green space Sidewalk coverage	Intersection density Green space Sidewalk density
Destination accessibility	Job within buffer	–
Distance to transit	Distance to nearest transit stop	Public transport density
Demand management	Parking supply and costs	–

#### GIS processing

For this analysis, population density was defined as the number of inhabitants per hectare. Densely populated areas encourage walking over driving due to accessibility of utilitarian destinations [43] and avoidance of traffic congestion [44]. Gridded population density data were obtained from Statistics Netherlands (CBS Statline) for the year 2018. Land use mix was measured using the land use entropy index, which captures how evenly land uses classes are distributed. The values range from 0 to 1 with 1 representing perfect mix of all relevant land uses [12]. A mixed land use is theorized to enhance walking since more desirable destinations are close by [11, 14]. For this analysis, we obtained Dutch land use dataset for 2015 from the National Georegister and included the following five land use categories in calculating land use mix: 1) commercial (retail and catering); 2) socio-cultural services (school, universities, hospitals and medical services, museums and concert halls); 3) residential areas; 4) offices and public services; and 5) green space and recreation (parks and recreation areas, sports and leisure activity areas). These land use classes have been identified to be relevant for walking [45]. Density of retail and service destinations were defined as the area proportion devoted to two land use classes “commercial” and “socio-cultural services” in each analysis unit for 2015. Street connectivity was defined as the point density of true intersections (i.e., three or more legs) on road segments that are accessible for pedestrians (e.g., excluding highways). A higher density of intersections is thought to be correlated with more walking through increasing the number of choices for getting to a destination on foot [15]. Intersections were derived from topographical maps of the Netherlands (TOP10 NL) for the year 2019. These data were obtained from data service of ESRI the Netherlands. Green space density was defined as the proportion of land devoted to parks, public gardens, forests and graveyards. Green space is sometimes classified as part of the natural environment rather than built, however, in urban settings green structures are more often planned and built as well. Green space data were obtained from the Dutch land use dataset 2015. Sidewalk density was defined as the area proportion of sidewalk. The rationale to include sidewalks as part of walkability is that it represents the availability of dedicated walking space, thus the safety aspect of walking for both transport and leisure purposes. Sidewalk data were obtained from the 2019 topographic map via ESRI the Netherlands. Public transport density was defined as the point density of all trams, buses, metros and ferries for short-range transport combined with density of train stations for long distance transport in 2018. Recent evidence showed that it promotes high frequency and longer distance of active transport modes, thereby increasing overall walking and also cycling in the Dutch context [23, 46, 47]. Public transport data were obtained from Geographic service of the University of Groningen (Geodienst Rijksuniversiteit Groningen, Groningen, the Netherlands). All geographical data for the seven components of the index were centralised, operationalised, and provided by the Geoscience and Health Cohort Consortium (GECCO) [48, 49].

To calculate walkability around the home environment, we applied circular (Euclidian) buffers centred around the geographical centre (centroid) of respondents’ residential six-digit postal code (PC6) area for every component. Euclidian buffer sizes were used for three reasons: first, a comparative Dutch study with green space suggest that Euclidian buffers result in more consistent associations with PA [50]. Second, Euclidian buffers do not require street network details and can thus be easily applied and adapted to other settings [51]. Third, we used circular buffers to be consistent with previous Dutch walkability studies [25, 28]. While there currently is no general consensus on which buffer sizes are most relevant for walking [10], especially not for the Dutch context, we applied three different sizes to estimate walkability in the immediate, medium and larger environments - corresponding to 150 m, 500 m, and 1000 m radii respectively. Walkability components were also calculated at administrative neighbourhood levels, as done in other walkability studies [28, 41]. In order to do this, raw componential data were first rasterized into 25 m × 25 m cells. For each raster cell, focal statistics were calculated for each of the three buffers and assigned to the PC6 addresses accordingly. To calculate neighbourhood walkability components, zonal statistics were employed to aggregate cell values to administrative neighbourhood boundaries.

#### Index calculation

Finally, we scaled these components by z-standardization such that they all have a mean of 0 and standard deviation of 1. Walkability index was then calculated by averaging the z-scores of these seven components. The resulting walkability index was then min-max scaled such that it ranged from 0 to 100 to help with interpretation of analysis results. The walkability index was composed for every PC6 area in 2017 in the Netherlands (*n* = 458,112) and subsequently linked to the OViN participants’ home PC6.

#### Walking as outcome

We analysed walking in the working adult population (from 18 to 65 years old) in the OViN 2017 survey (*n* = 16,055) since children and older adults likely have different mobility patterns and dependency on the environment and thus results cannot easily be generalized for the entire population [52]. We also excluded those who did not report mobility due to personal circumstances (being sick, having disabilities, work/study, being on vacation, having no activity outside planned, weather condition) as these do not reflect daily patterns of mobility. We also excluded those who travelled outside the Netherlands since we assumed that trips to other countries did not belong to a daily mobility pattern.

For each participant, primary outcome variables computed were absolute distance walked (in meters) and time spent walking (in minutes) per day, regardless whether the trips were made with single or multiple transport means. We theorized that walkability around residential neighbourhoods initiated walking at both starting point and destination due to cascading modal choices. For example, when residential neighbourhoods are walkable; instead of taking the car to a specific location such as the (super)market, residents could choose to walk and/or utilize public transit and from there continue with active transport to other places. Therefore, total walking duration or distance was a relevant outcome.

Secondary outcome variables were time spent walking for specific purposes. We dichotomized the purpose classes based on Frank et al. (2003), which defined discretionary trips as those for leisure (non-commuting) and non-discretionary as those for transport-related (commuting) and essential shopping purposes [53]. Since the transport survey did not make a distinction between groceries and leisure shopping, we further modified the discretionary/ nondiscretionary dichotomy additionally based on availability of choices: whether the respondent could choose between multiple locations for the same purpose. Work and study trips are non-discretionary because these are fixed trips whose destinations respondents had little to no control over; whereas grocery and shopping trips are discretionary where multiple choices for supermarkets and shopping malls exist. Other survey choices including leisure walks, sports and hobbies, recreational visits to restaurants and other service destination and home visit also fall under discretionary purpose.

#### Covariates

We adjusted the analyses for a predefined set of confounders including age groups (18–35, 36–49, 50–65 years old), sex, ethnic background (native Dutch, non-Dutch Western, and Non-Western), highest education obtained (low, medium or high), work status (study, fulltime work, part-time work, not working i.e. including early retirees), standardized household income (low, medium or high), household car ownership (no car, one car, two or more cars), household composition (single, couple, couple with children, single parent with children, others) and whether respondents also cycled on the same day; and survey-related confounders such as seasonality (spring, summer, autumn or winter), day of the week (weekday or weekend), survey response type (face-to-face, online or via telephone) and neighbourhood SES score. We utilized the SES score created by the Netherlands Institute for Social Research (SCP). This SES score was a continuous variable based on (1) the average income, (2) the percentage of residents with a low income, (3) the percentage of residents with a low level of education, and (4) the percentage of unemployed residents in the neighbourhood [54]. A higher score indicated higher neighbourhood-level SES.

#### Statistical analysis

Descriptive statistics on individual and neighbourhood characteristics of respondents were summarized as percentage for categorical variables, mean (standard deviation) or median [interquartile range] for continuous variables – dependent on distribution of the variable - for the full sample and per quintile of walkability. Pearson correlation was computed for pairwise correlation between any two components, as well as between each component and the resulting index. Due to the relatively small number of missing data (< 10%), we performed complete case analyses for all models presented in this study.

More than half of the respondents did not report any walking activity on their survey date, resulting in a large percentage of zero values in all walking outcomes. This left-censored data structure ruled out ordinary least square regression due to violation of basic assumptions. Instead, we used Tobit models, an increasingly common modelling framework in recent transport studies [55–57] to analyse the associations between walkability and walking duration and distance. In short, Tobit models provide similar interpretation to regular linear regression; however, effect estimates are conditional on probability that walking outcome is above zero. While excluding respondents who did not walk might reduce bias in effect estimate, Lachapelle and Jean-Germain (2019) advocated the use of Tobit models to prevent overestimation of effect sizes [56]. Since the number of neighbourhoods far exceeded the number of respondents and there was at most one respondent per PC6 area, we did not cluster observations or carry out multilevel modelling.

In primary analyses, model outcomes were presented as an effect estimate and 95% confidence interval (CI) for differences in average walking times (in minutes) and distances (in meters) per 10% increase in walkability. Two models were presented: Model 1 is an unadjusted model and Model 2 is a fully adjusted model that included all covariates mentioned in 3.1. We also stratified the walking outcomes by purpose: discretionary and non-discretionary.

For secondary analysis, we investigated which walkability components mostly drove the association with walking outcomes. We did this by regressing six-component indices, thus leaving one component out at a time, against time spent walking using fully adjusted Tobit models. If significant attenuation of the effect estimates association was observed in any of these partial indices, we concluded the missing component to be driving the association in the main seven-component index [58].

As a tertiary analysis, we examined whether certain sociodemographic characteristics and urbanicity modified the associations between walkability and walking outcomes. For this purpose, we conducted stratified analyses on sex, age groups (18–35, 36–49 and 50–65 years old), neighbourhood SES (lowest, middle and highest tertile) and degrees of urbanization (< 1000, 1000–2500 and > 2500 addresses/km^2^) regardless of significance of the interaction term. All data analyses were carried out in R (R Core Team, Vienna, Austria) using package *censReg* for Tobit modelling [59, 60].

## Results

### Walkability index

**Fig. 1:**
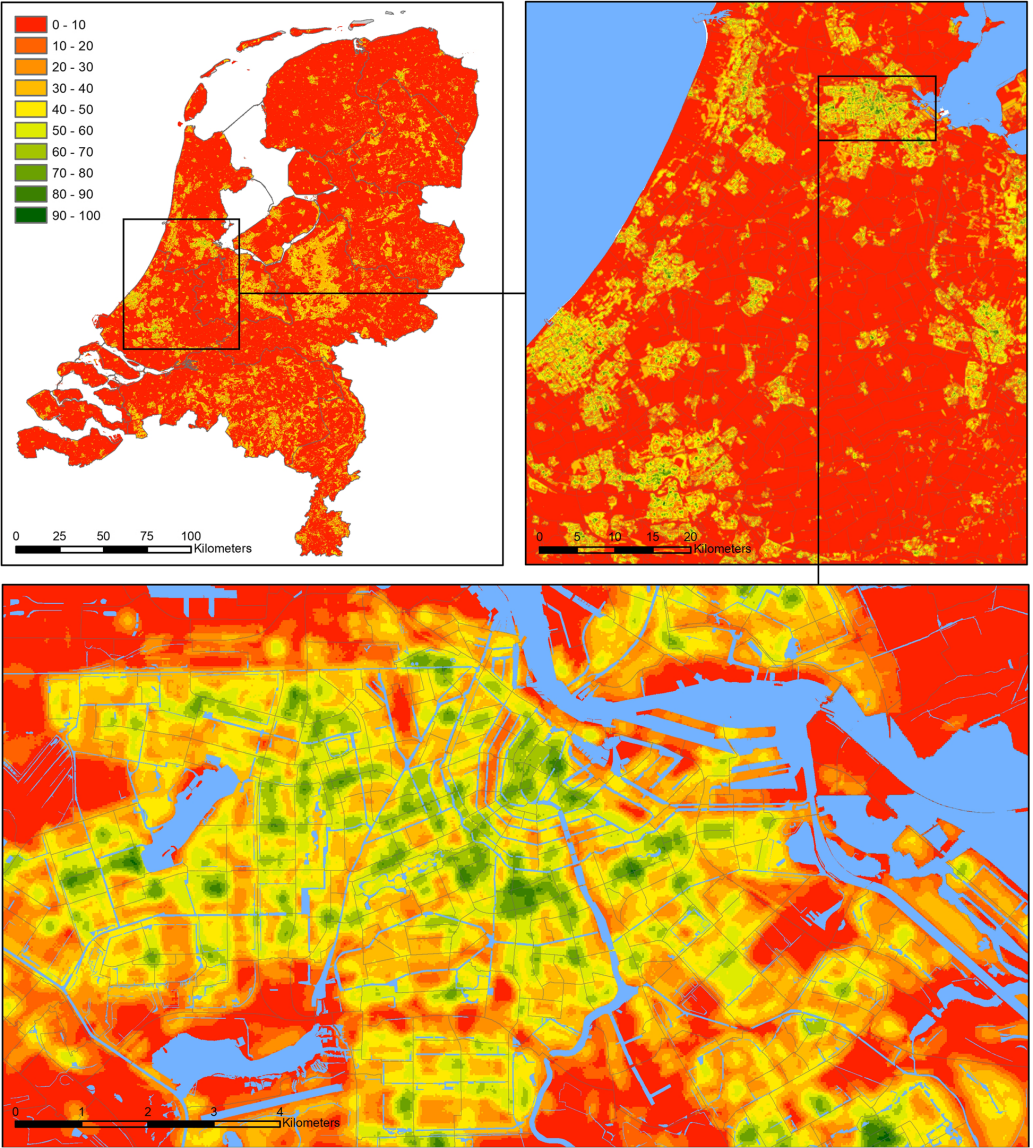
Walkability index map 150 m buffer size for the Netherlands (top left), the densely populated region of Randstad (top right) and the city of Amsterdam (bottom). Walkability is scaled from 0 to 100 where red denotes the 10% lowest walkability scores, and green denotes the 10% highest walkability scores

**Fig. 2:**
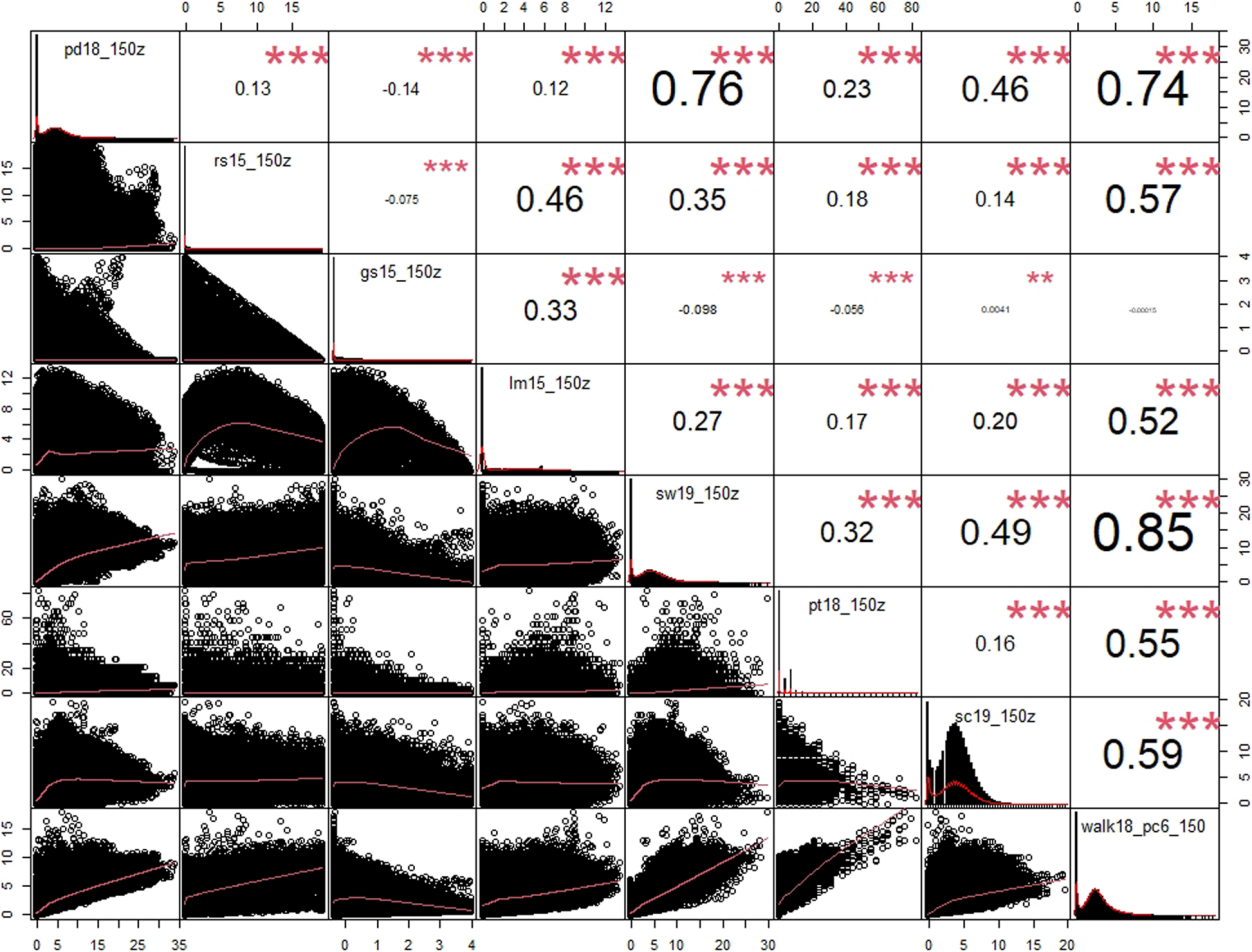
Pearson correlation matrix for walkability components of 150 m buffer for all PC6 addresses in the Netherlands. The top part of the matrix denotes the absolute value of correlation and significance levels (*** denotes *p*-value < 0.001, ** for *p*-value < 0.01). The bottom half denotes bivariate scatterplots between two corresponding variables with a fitted line showing direction of correlation. pd18_150z: population density, rs15_150z: retail & service destination density, gs15_150z: green space, lm15_150z: land use mix, sw19_150z: sidewalk density, pt18_150z: public transport density, sc19_150z street connectivity, walk18_pc6_150: walkability index

The walkability index was composed for all 458,112 PC6 areas in the Netherlands. The most walkable area was in the city centre of the Hague whereas the least walkable area was found to be in the Port of Amsterdam. Highly walkable areas clustered in the centre of major cities in the Netherlands as exemplified by the city of Amsterdam, where walkability is highest in the city centre and gradually reduce towards the outskirts (Fig. 1). Nationwide, the most walkable areas were therefore found in the Randstad, a region consisting of the four largest Dutch cities and their surroundings (Fig. 1). The seven components of the index were moderately correlated with each other, with the highest R-value of 0.76 between population density and sidewalk density (Fig. 2).

### Descriptive statistics

Of the 16,055 respondents, there were slightly more females, but respondents were rather evenly distributed between the age groups across quintiles of walking (Table 2). Most of the respondents had native Dutch background, were medium- to highly educated and had at least one car in the household. Approximately half of the respondents worked fulltime, had high income or had at least one child. On average, respondents walked 9 min - or 744 m - per day, even though the median value for both variables is 0 meaning more than half of the included respondents did not walk at all on sampled date. When examined across the quintiles of neighbourhood walkability, there was a trend towards higher percentage of non-native Dutch, high education attainment, lower number of cars, higher share of single-person households, higher urbanization degrees and decreasing neighbourhood SES score with increased walkability.

**Table 2:** Socio-demographic characteristics of the OViN respondents by quintiles of neighbourhood walkability. Categorical data were presented by count (percentage) and continuous data presented as mean (SD) or median [IQR]

Quintiles of walkability	1 (lowest)	2	3	4	5 (highest)	Overall
Number of respondents	3120	3203	3238	3275	3219	16,055
Female	1616 (51.8%)	1687 (52.7%)	1697 (52.4%)	1721 (52.5%)	1706 (53.0%)	8427 (52.5%)
Age group						
18–35	944 (30.3%)	933 (29.1%)	938 (29.0%)	1048 (32.0%)	1345 (41.8%)	5208 (32.4%)
36–49	905 (29.0%)	1001 (31.3%)	1027 (31.7%)	1070 (32.7%)	940 (29.2%)	4943 (30.8%)
50–65	1271 (40.7%)	1269 (39.6%)	1273 (39.3%)	1157 (35.3%)	934 (29.0%)	5904 (36.8%)
Ethnic background						
Native Dutch	2909 (93.2%)	2818 (88.0%)	2716 (83.9%)	2564 (78.3%)	2314 (71.9%)	13,321 (83.0%)
Other Western	154 (4.9%)	234 (7.3%)	292 (9.0%)	306 (9.3%)	359 (11.2%)	1345 (8.4%)
Non-Western	57 (1.8%)	151 (4.7%)	230 (7.1%)	405 (12.4%)	546 (17.0%)	1389 (8.7%)
Highest education obtained						
Low	86 (2.8%)	64 (2.0%)	112 (3.5%)	117 (3.6%)	129 (4.0%)	508 (3.2%)
Medium	1940 (62.2%)	1942 (60.6%)	1813 (56.0%)	1853 (56.6%)	1508 (46.8%)	9056 (56.4%)
High	1094 (35.1%)	1197 (37.4%)	1313 (40.5%)	1305 (39.8%)	1582 (49.1%)	6491 (40.4%)
Work status						
Work part-time	727 (23.3%)	818 (25.5%)	672 (20.8%)	670 (20.5%)	534 (16.6%)	3421 (21.3%)
Work fulltime	1697 (54.4%)	1695 (52.9%)	1795 (55.4%)	1805 (55.1%)	1813 (56.3%)	8805 (54.8%)
Student	213 (6.8%)	192 (6.0%)	247 (7.6%)	274 (8.4%)	332 (10.3%)	1258 (7.8%)
Not working	483 (15.5%)	498 (15.5%)	524 (16.2%)	526 (16.1%)	540 (16.8%)	2571 (16.0%)
Standardized household income						
Low	365 (11.7%)	461 (14.4%)	540 (16.7%)	657 (20.1%)	856 (26.6%)	2879 (17.9%)
Medium	992 (31.8%)	1051 (32.8%)	1009 (31.2%)	1056 (32.2%)	939 (29.2%)	5047 (31.4%)
High	1763 (56.5%)	1691 (52.8%)	1689 (52.2%)	1562 (47.7%)	1424 (44.2%)	8129 (50.6%)
Household car ownership						
No car	89 (2.9%)	159 (5.0%)	311 (9.6%)	405 (12.4%)	927 (28.8%)	1891 (11.8%)
One car	1063 (34.1%)	1401 (43.7%)	1524 (47.1%)	1701 (51.9%)	1607 (49.9%)	7296 (45.4%)
Two or more cars	1968 (63.1%)	1643 (51.3%)	1403 (43.3%)	1169 (35.7%)	685 (21.3%)	6868 (42.8%)
Household situation						
Single-person household	278 (8.9%)	390 (12.2%)	477 (14.7%)	552 (16.9%)	906 (28.1%)	2603 (16.2%)
Couple without children	914 (29.3%)	860 (26.8%)	821 (25.4%)	819 (25.0%)	824 (25.6%)	4238 (26.4%)
Couple with children	1760 (56.4%)	1701 (53.1%)	1656 (51.1%)	1580 (48.2%)	1100 (34.2%)	7797 (48.6%)
Single parent with children	136 (4.4%)	225 (7.0%)	245 (7.6%)	258 (7.9%)	232 (7.2%)	1096 (6.8%)
Other compositions	32 (1.0%)	27 (0.8%)	39 (1.2%)	66 (2.0%)	157 (4.9%)	321 (2.0%)
Season						
Spring	846 (27.1%)	845 (26.4%)	824 (25.4%)	825 (25.2%)	835 (25.9%)	4175 (26.0%)
Summer	906 (29.0%)	914 (28.5%)	945 (29.2%)	927 (28.3%)	860 (26.7%)	4552 (28.4%)
Autumn	692 (22.2%)	712 (22.2%)	729 (22.5%)	734 (22.4%)	733 (22.8%)	3600 (22.4%)
Winter	676 (21.7%)	732 (22.9%)	740 (22.9%)	789 (24.1%)	791 (24.6%)	3728 (23.2%)
Urbanization degree						
> 2500 addresses/km^2^	63 (2.0%)	224 (7.0%)	387 (12.0%)	980 (29.9%)	2050 (63.7%)	3704 (23.1%)
1000–2500	952 (30.5%)	1514 (47.3%)	2171 (67.0%)	2025 (61.8%)	1079 (33.5%)	7741 (48.2%)
< 1000	2105 (67.5%)	1465 (45.7%)	680 (21.0%)	270 (8.2%)	90 (2.8%)	4610 (28.7%)
Day of the week: Weekend	805 (25.8%)	818 (25.5%)	832 (25.7%)	841 (25.7%)	850 (26.4%)	4146 (25.8%)
Response type						
Internet	1220 (39.1%)	1249 (39.0%)	1202 (37.1%)	1242 (37.9%)	1192 (37.0%)	6105 (38.0%)
Telephone	1164 (37.3%)	1129 (35.2%)	1132 (35.0%)	1051 (32.1%)	836 (26.0%)	5312 (33.1%)
Face-to-face	736 (23.6%)	825 (25.8%)	904 (27.9%)	982 (30.0%)	1191 (37.0%)	4638 (28.9%)
Respondents also bike on the same day						
Yes	599 (19.2%)	786 (24.5%)	825 (25.5%)	866 (26.4%)	1016 (31.6%)	4092 (25.5%)
Neighbourhood socioeconomic status score						
Mean (SD)	0.126 (0.864)	0.0447 (1.03)	0.0435 (1.13)	−0.151 (1.24)	−0.283 (1.31)	−0.0453 (1.14)
Median [IQR]	0.180 [0.950]	0.100 [1.27]	0.160 [1.46]	0.0600 [1.65]	−0.190 [1.69]	0.0900 [1.38]
Total time spent walking, minutes per day						
Mean (SD)	6.04 (20.3)	7.81 (21.2)	8.49 (22.5)	8.93 (23.4)	12.2 (33.2)	8.72 (24.6)
Median [IQR]	0 [0]	0 [0]	0 [0]	0 [7.00]	0 [13.0]	0 [4.00]
Total distance walked, meters per day						
Mean (SD)	543 (1830)	690 (1910)	709 (1910)	795 (2020)	975 (2280)	744 (2000)
Median [IQR]	0 [0]	0 [0]	0 [0]	0 [500]	0 [1000]	0 [400]
Discretionary walk time (minutes) per day						
Mean (SD)	5.07 (19.4)	6.44 (20.3)	7.04 (21.4)	6.90 (22.1)	8.78 (25.9)	6.86 (22.0)
Median [IQR]	0 [0]	0 [0]	0 [0]	0 [0]	0 [5.00]	0 [0]
Non-discretionary walk time (minutes) per day						
Mean (SD)	0.969 (5.92)	1.37 (6.65)	1.45 (7.23)	2.03 (8.52)	3.45 (21.5)	1.86 (11.6)
Median [IQR]	0 [0]	0 [0]	0 [0]	0 [0]	0 [0]	0 [0]

### Association with walking

Each 10% increase in walkability at 150 m buffer corresponded with 8.5 min (95% CI: 7.0–9.9) or 630 m (95% CI: 510–750) increase in walking after adjustment for all confounders. There are clear and consistent associations with increased walking across all buffer sizes studied and also for administrative neighbourhood boundaries (Table 3). Since the association with 150 m buffer size has the highest effect estimates of all scales studied, we evaluated secondary analyses based on 150 m buffer size index.

**Table 3:** Censored regression models for associations between walkability around home address and time spent walking or distance walked. The effect estimates for walkability measures were presented, denoting effect estimate per 10% increase in national walkability indices of different buffer sizes and 95% confidence interval. *N* = 16,055

		Neighbourhood walkability	150 m buffer walkability	500 m buffer walkability	1000 m buffer walkability
Total distance walked (meters)	Model 1	810 (710, 900)	970 (860, 1080)	750 (670, 830)	620 (550, 690)
	Model 2	520 (410, 620)	630 (510, 750)	520 (430, 600)	420 (340, 490)
Non-discretionary distance walked (meters)	Model 1	890 (760, 1010)	1020 (870, 1160)	810 (710, 910)	690 (600, 790)
	Model 2	470 (340, 610)	540 (390, 690)	470 (360, 590)	400 (300, 500)
Discretionary distance walked (meters)	Model 1	700 (590, 810)	870 (740, 990)	650 (560, 750)	530 (450, 610)
	Model 2	500 (380, 620)	650 (510, 780)	500 (400, 600)	390 (300, 480)
Total time walked (minutes)	Model 1	10.3 (9.2, 11.5)	12.5 (11.1, 13.8)	9.6 (8.7, 10.6)	7.9 (7.1, 8.7)
	Model 2	6.8 (5.6, 8.1)	8.5 (7.0, 9.9)	6.8 (5.7, 7.9)	5.6 (4.7, 6.5)
Non-discretionary time walked (minutes)	Model 1	10.7 (9.2, 12.2)	12.4 (10.7, 14.1)	9.8 (8.6, 11.1)	8.4 (7.3, 9.4)
	Model 2	5.8 (4.2, 7.4)	6.9 (5.1, 8.7)	6.0 (4.6, 7.3)	5.0 (3.8, 6.1)
Discretionary time walked (minutes)	Model 1	9.1 (7.7, 10.4)	11.1 (9.6, 12.7)	8.4 (7.3, 9.5)	6.7 (5.7, 7.7)
	Model 2	6.7 (5.2, 8.1)	8.5 (6.8, 10.2)	6.5 (5.3, 7.8)	5.2 (4.1, 6.3)

Model 1 denotes unadjusted models (only walkability index and outcome)

Model 2 denotes fully-adjusted models (confounders include age, sex, ethnic background, education, work status, household standardized income group, neighbourhood SES, car possession, household situation, seasonality, weekday or weekend, response type and whether respondents also biked on the same day)

### Discretionary vs. non-discretionary walking

Each 10% increase in walkability at 150 m buffer was slightly more strongly associated with discretionary walking (effect estimates for distance: 650 m, 95%CI: 510–780 and for time 8.5 min, 95%CI: 6.80–10.2) than non-discretionary walking (effect estimates for distance: 540 m, 95%CI: 390–690 and for time 6.9 min, 95%CI: 5.1–8.7) even though the confidence intervals did overlap. Overall, associations were consistently and statistically significantly associated with increased walking across all buffer sizes and walking outcomes, before and after adjustment for confounders (Table 3).

### Influential components

Leaving out public transport density strongly attenuated the association with walking by lowering all effect estimates by almost half, e.g. 5.2 min walked per 10% increase in walkability at 150 m buffer (95%CI: 4.3–6.1), suggesting that public transport density was the strongest driver of the index at 150 m buffer size (Table 4).

**Table 4:** Censored regression model for associations between leave-one-out walkability indices (composed for 150 m buffer around home address) and time spent walking. The effect estimates for walkability measures are presented, denoting effect estimate per 10% increase in full and partial walkability indices and 95% confidence interval in fully adjusted models (Model 2). *N* = 16,055

	Total time walked (minutes)	Discretionary time walked (minutes)	Non-discretionary walk time (minutes)
Walkability index 150 m buffer size	**8.5** **(7.0, 9.9)**	**8.5** **(6.8, 10.2)**	**6.9** **(5.1, 8.7)**
Population density	9.5 (7.7, 11.3)	9.4 (7.5, 11.6)	7.8 (5.5, 10.0)
Retail & service destination density	9.4 (7.8, 11.1)	9.5 (7.6, 11.4)	7.5 (5.4, 9.60)
Land use mix	8.7 (7.2, 10.2)	8.7 (6.9, 10.5)	7.2 (5.3, 9.2)
Street connectivity	9.5 (7.9, 11.1)	9.5 (7.6, 11.4)	7.7 (5.6, 9.7)
Green space	8.8 (7.3, 10.3)	8.8 (7.1, 10.5)	7.2 (5.3, 9.1)
Sidewalk density	8.2 (6.7, 9.8)	8.6 (6.8, 10.4)	6.4 (4.4, 8.3)
Public transport density	**5.2** **(4.3, 6.1)**	**5.1** **(4.0, 6.1)**	**4.4** **(3.2, 5.5)**

### Effect modifiers

We only reported secondary and tertiary research analyses for 150 m buffer size since primary analyses with this buffer size showed the strongest association with walking outcome. Stratified analysis results showed that associations generally differed across urbanization and demographic strata, but interaction terms were only statistically significant in non-discretionary walking (Table S1). Associations with minutes of non-discretionary walking were stronger in rural (11.0, 95%CI: 6.4–15.6) compared to urban areas (3.3, 95%CI: 0.9–5.6); in low neighbourhood SES (9.9, 95%CI 6.1–13.8) compared to high (3.2, 95%CI 0.8–5.6); in middle-aged adults (36–50 years, 12.4, 95%CI 7.0–17.7) and older adults (51–65 years, 8.9, 95%CI: 5.8, 11.9) compared to younger adults (18–35 years old, 3.3, 95%CI: 1.4–5.1) (Table 5). The interaction term for sex was not statistically significant (Table S1), which was also evident in stratified analysis for males (8.7, 95%CI: 5.6–11.7) versus females (4.8, 95%CI 2.9–6.8). Furthermore, the association between walkability and non-discretionary walking in highly urban areas was not significant. Censored regression analysis including the interaction terms for all these effect modifiers were reported in Table S1.

**Table 5:** Censored regression model for associations between walkability index at 150 m buffer around home address and time spent walking across different strata. The effect estimates for walkability measures are presented, denoting effect estimate per 10% increase in walkability indices and 95% confidence interval. Here only full-adjusted model results were presented (confounders include age (not included when age was stratified), sex (not included when sex was stratified), ethnic background, education, work status, household standardized income group, neighbourhood SES (not included when SES was stratified), car possession, household situation, seasonality, weekday or weekend, response type and whether respondents also biked on the same day). *N* = 16,055. For neighbourhood SES strata, the median [IQR] was provided

Stratum	N	Total time walked (minutes)	Discretionary time walked (minutes)	Non-discretionary time walked (minutes)
**Highly urban**	3704	5.1 (2.5, 7.8)	5.5 (2.7, 8.3)	3.0 (−1.0, 6.9)
**Urban**	7741	7.2 (4.8, 9.6)	9.1 (6.1, 12.1)	3.3 (0.9, 5.6)
**Rural**	4610	10.4 (6.7, 14.2)	9.1 (4.7, 13.5)	11.0 (6.4, 15.6)
**Low neighbourhood SES** 16 [11.6]	5390	8.5 (6.1, 10.9)	7.2 (4.6, 9.7)	9.9 (6.1, 13.8)
**Middle SES** 13 [11.8]	5339	9.0 (6.6, 11.5)	9.3 (6.4, 12.3)	6.4 (3.8, 9.0)
**High SES** 13.7 [11.4]	5326	7.6 (5.1, 10.1)	9.1 (6.0, 12.3)	3.2 (0.8, 5.6)
**Male**	7628	8.5 (6.4, 10.6)	7.7 (5.4, 10.1)	8.7 (5.6, 11.7)
**Female**	8427	8.1 (6.2, 10)	8.7 (6.4, 11.0)	4.8 (2.9, 6.8)
**18–35 years old**	5208	6.6 (4.6, 8.5)	8.1 (5.6, 10.7)	3.3 (1.4, 5.1)
**36–49 years old**	4943	8.7 (5.7, 11.7)	6.4 (3.4, 9.4)	12.4 (7.0, 17.7)
**50–65 years old**	5904	10.1 (7.5, 12.8)	9.5 (6.4, 12.6)	8.9 (5.8, 11.9)

## Discussion

### Main findings

We constructed a theory-driven, evidence-informed nationwide walkability index for the Netherlands. More walkable neighbourhoods were consistently associated with increased total walking duration and distance, regardless of buffer sizes and boundaries used. In terms of components, public transport density was most strongly associated with walking outcomes. Furthermore, stratified analyses showed that the association between walkability and non-discretionally walking was especially stronger in middle-aged and older adults compared to young, those who lived in rural compared to urban; and those in neighbourhoods with low SES scores compared to high SES.

### Interpretation

To our knowledge, this study was one of the very few that examined walkability index at fine geographical scales for an entire country. We observed consistent associations between walkability indices of different buffer sizes (150 m, 500 m and 1000 m) and administrative neighbourhood-level walkability with time spent walking, either total or purpose-specific. This in in line with findings from the four-component American National Walkability Index by the Environmental Protection Agency [61]. This index consisted of residential density, intersection density, distance to public transport and land uses relating to employment and household types; and was calculated at block group level. Watson and colleagues (2020) found that living in the most walkable areas is association to an 10% increase in geometric mean walking time for leisure and up to 26% walking time for transportation [62]. While there are some studies assessing walking-related built environmental factors separately, only one other study reported the construction of a walkability index for the Netherlands. Liao et al. (2020) employed a regression-based, data-driven approach to construct a walkability index which eventually consisted of total inland water, distance to supermarket, number of daily goods stores within 1 km, number of cafeterias within 1 km, land use for residential buildings and high urban density [41]. Even though the components did not overlap, the resulting walkability maps were similar in indicating higher walkability in more urban areas. This similarity also suggests that for the Dutch context, environmental factors that facilitate utilitarian walking are important.

Walkability at 150 m buffer size had the largest effect size, which slightly declined with increasing buffer sizes. This finding was also reported by an earlier study carried out in Australian adults in Perth, where walkability was considered relevant especially in smaller -but also in larger- buffers [63]. This may indicate that either the immediate environmental factors play a more important role in influencing walking behaviours or there was larger variance in home walkability of participants in the smaller buffer sizes.

Our componential results showed that public transport density was the most influential component of the index, where associations reduced almost by half upon its exclusion. While public transport density is not a commonly used component of walkability, our study results suggest that it significantly improved the index association with walking, especially in discretionary walking. Gao and colleagues (2020), who investigated walkability components and walking for different purposes using the same transport survey as the current study, also reported that public transport users tended to walk more than non-users, which explains how adding public transport density significantly increased effect sizes of walkability in our models [23]. A multi-cohort study in Germany also found public transport to be associated with walking and cycling in their participants, besides street connectivity and destination density [64]. The Netherlands has an extensive system of public transport which is characterized by high density in urban and efficiently run in rural areas. Especially in rural areas, public transport means are interlinked to facilitate transit, and buses are flexible, some of which operate based on customers’ demands. This ensures a high level of service while maintaining efficiency [65]. Furthermore, Daniels and Mulley (2013) found in their Sydney-based study that the different modes of transport available near the home could potentially influence walking distance: participants tend to walk more to access a train than a bus [47]. In future walkability studies it would therefore be beneficial to also examine short- and long-distance transport stops separately.

Stratified analysis showed that significant effect modification by age, urbanization degrees or neighbourhood SES was observed mostly in work- and study-related walking. In particular, associations with non-discretionary walking were higher in rural than urban areas, in low SES neighbourhoods than high, and in the middle- and older- than young adults. Associations with discretionary walking, on the other hand, did not differ significantly across these strata. The urban rural discrepancy was observed in the American index; however, the direction was reverse: associations between walkability and walking for transportation and leisure in the American study were only significant for urban and not rural areas. While the US Census dichotomized definition of rural and urban is slightly different from the urbanization grades used in this study [66], there was a clear discrepancy in rural versus urban walkability between the two countries. 65% of participants in rural America lived in the least walkable and 0% in the most walkable category, while only 29% of the participants in the lowest quintile of walkability and 28% in the highest quintile of walkability live in Dutch rural areas (Table 2). The lack of variance in walkability in rural America might drive the lack of associations with walking outcome, while for the Dutch context, walkability was much more evenly distributed. For neighbourhood SES, Adkin et al. (2017) conducted a literature review and found that associations between built environment and walking were generally weaker for disadvantaged groups compared to advantaged groups across all domains of walking (transport, leisure and PA), which contrasted our findings [30]. In the Dutch context, this result might be explained by residents in the lower SES neighbourhoods possessing fewer cars and they are therefore more reliant on active transport modes such as walking, cycling and public transit to travel to work.

### Strengths and limitations

An important strength of our study is that we were able to construct a high-resolution nationwide index consisting of seven objectively measured built environmental components on a detailed spatial scale, capturing multiple aspects of the built environment relevant for walking. Compared to early versions of walkability index, our walkability index is more comprehensive since it incorporates recently studied components such as green space, which is often referred to in other studies as “green walkability” [67] or “eco-friendly walk score” [68]; and public transport density as included in US EPA’s national walkability index [61]. By examining this index using different buffer sizes and boundaries, we could assess the sensitivity of the association, thereby partially mitigating scale and zoning effects. Moreover, we were able to utilize a well-established travel survey for validation of the index, which is representative of the Dutch population in terms of age, geographical distribution and also travel pattern throughout the year.

Our study also has some limitations to consider. Firstly, some potentially important aspects of the built environment relevant for walking were not included because they were unavailable or the quality was not high, such as aesthetics or safety [39]. Secondly, since this was a cross-sectional study, no causal relation could be inferred. Information on residential self-selection was not collected in the survey and we can thus not rule out reverse causation where active participants chose to live in more walkable neighbourhoods rather than the environmental walkability determining level of walking. Thirdly, the various components in the index were combined based on equal weighting, which might not capture the relative importance of these components for walking. For example, a parcel-based 3D walkability index developed for Seattle and Baltimore [69] weighted street connectivity more than other components to better reflect where more walking was reported. Data-driven methods to weigh components such as regression with walking time or distance as outcome or utilizing analytic hierarchy process could further contextualize the walkability index but in turn reduce its comparability with international studies. Lastly, OViN as a survey instrument suffers from some forms of biases: since all walking data were self-reported which might be prone to recall bias. Moreover, one study found that compared to a place-based survey, OViN respondents were likely to underreport short trips, cycling and walking trips and, non-home-based trips [70]. This place-based approach thus has replaced trip-based approach for reporting mobility in the survey from 2018 onwards [71].

### Suggestions for future research

Studies with walkability indices in the Netherlands are still in its infancy. Although only a handful applications are published so far, they started to explore relevant aspects that should be deepened. Firstly, incorporation of walkability at destinations (such as work) outside of the home could provide some additional insights into transport mode choice. Moreover, in the Dutch context, it would be interesting to also examine whether walkability index is also associated with cycling, as demonstrated in an earlier Austrian study [21] or whether a separate cycleability index is necessary. In terms of index composition methodologies, a combination of both theory- and data-driven methodologies could be the way forward to balance between contextualization and conceptualization of walkability index. Moving forward, methods that integrate objective and subjective measures could be worth examining. For instance by placing the pedestrian in the centre of the walking experience and capturing both subjective assessment of the walking experience in real time, using biosensors and the objective assessment of walking infrastructure at high spatial resolutions [72]. In terms of mobility measurement, other objective instruments such as public transport cards, social media, GPS trackers and location trackers are promising for local use [73]; however, these are also associated with high costs data collection, intensive data processing, privacy concerns and response burden. To improve causal inference, studies exploring walkability and PA in longitudinal cohorts could be considered. Given the validity of our current walkability index, future studies could examine the associations between walkability and other health outcomes further downstream.

### Implications for practice

Our walkability index can be useful for policy makers and urban planners to characterize, benchmark and monitor walkability in the Netherlands thereby identifying potential targets for improvement. Currently, walkability data are available from as early as 1989 and can be regularly updated, enabling both prospective and retrospective examinations of walkability alone or with health outcomes. In terms of monitoring, limited evidence on longitudinal walkability showed that changes in walkability index and its components were minimal (and not statistically significant) between 2005, 2008 and 2011 [25]; which suggests that monitoring period should be longer than the examined period in that study.

Moreover, this study has raised important policy implications: the rural and low SES neighbourhood residents would benefit more from increased walkability. Especially walking as a form of active commuting for work- and study-related purposes could therefore be prioritized for interventions. Currently, 65% of the Dutch population are between 15 and 64 years old, the legal working age, so even a small increase in walking per day would already have large effect on public health outcomes. Moreover, given that public transport density is a significant contributor to walkability, improving the public transport network could be prioritized in order to promote walking in low walkability areas.

## Conclusions

Our nationwide walkability index was associated with increased total and purpose-specific walking behaviours of adults in the Netherlands; especially in rural areas, low SES neighbourhoods, and middle-aged strata; indicating its value for further use in the Netherlands.

## Supplementary Information

**Additional file 1: Table S1.** Censored regression model for associations between walkability index at 150 m buffer around home address and time spent walking plus interaction terms. In this table, only *p*-values of the respective interaction terms are reported. Here only results from fully-adjusted models are presented (confounders include age, sex, ethnic background, education, work status, household standardized income group, neighbourhood SES, car possession, household situation, seasonality, weekday or weekend, response type and whether respondents also biked on the same day).

## Data Availability

Walkability data are available upon requests via GECCO website (www.gecco.nl). Transport survey (OViN) data are not publicly available at the resolution analysed in this study (6-digit postal code) due to regulations from the governing organization (Statistics Netherlands). Under certain conditions, these microdata are accessible for statistical and scientific research. For further information: microdata@cbs.nl. 4-digit postal code data are however publicly available and can be downloaded free of charge via website: https://easy.dans.knaw.nl/ui/datasets/id/easy-dataset:103498
