# Supplementary material for: Development of an objectively measured walkability index for the Netherlands
Source: Int J Behav Nutr Phys Act. 2022 May 2;19:50. doi: 10.1186/s12966-022-01270-8 (PMC9063284; doi:10.1186/s12966-022-01270-8)
Supplement: Supplementary file 1 — Additional file 1: Table S1. Censored regression model for associations between walkability index at 150 m buffer around home address and time spent walking plus interaction terms. In this table, only p-values of the respective interaction terms are reported. Here only results from fully-adjusted models are presented (confounders include age, sex, ethnic background, education, work status, household standardized income group, neighbourhood SES, car possession, household situation, seasonality, weekday or weekend, response type and whether respondents also biked on the same day). [file 12966_2022_1270_MOESM1_ESM.docx]

**Development and validation of an objectively measured walkability index for the Netherlands**

Thao Minh Lam^1*^, Zhiyong Wang^2^, Ilonca Vaartjes^3,4,5^, Derek Karssenberg^4,6^, Dick Ettema^2^, Marco Helbich^2^, Erik J Timmermans^3^, Lawrence Frank^7,8^, Nicole Den Braver^1^, Alfred Wagtendonk^1^, Joline WJ Beulens^1,3^, Jeroen Lakerveld^1^

*^1^ Amsterdam UMC, Vrije Universiteit Amsterdam, Department of Epidemiology and Data Science, Amsterdam Public Health research institute, De Boelelaan 1117, Amsterdam, the Netherlands.*

*^2^ Department of Human Geography and Spatial Planning, Faculty of Geosciences, Utrecht University, Princetonlaan 8a, 3584 CB Utrecht, Netherlands*

*^3^* Julius Center for Health Sciences and Primary Care, University Medical Center Utrecht, Utrecht University, Utrecht, Netherlands

*^4^* Global Geo Health Data Center, University Medical Center Utrecht & Utrecht University, Utrecht, Netherlands

*^5^* Dutch Health Foundation, The Hague, Netherlands

*^6^* Department of Physical Geography, Utrecht University, *Princetonlaan 8a, 3584 CB Utrecht, Netherlands*

*^7^* Department of Urban Studies and Planning, UC San Diego, La Jolla, San Diego, United States of America

*^8^* Urban Design 4 Health, Seattle, Washington, United States of America

**Corresponding author: Thao Minh Lam, (t.m.lam@amsterdamumc.nl) Department of Epidemiology and Data Science, Amsterdam University Medical Centers, location VUmc. De Boelelaan 1089a, 1081HV Amsterdam, the Netherlands*

**Supplementary table**

**Table S1.** Censored regression model for associations between walkability index at 150m buffer around home address and time spent walking plus interaction terms. In this table, only p-values of the respective interaction terms are reported. Here only results from fully-adjusted models are presented (confounders include age, sex, ethnic background, education, work status, household standardized income group, neighbourhood SES, car possession, household situation, seasonality, weekday or weekend, response type and whether respondents also biked on the same day) .

| **Interaction term** | **Total time walked (minutes)** | **Discretionary time walked (minutes)** | **Non-discretionary time walked (minutes)** |
| --- | --- | --- | --- |
| Highly urban | ref | ref | ref |
| Urban | 0.41 | 0.26 | 0.79 |
| Rural | 0.11 | 0.57 | **0.01** |
|  |  |  |  |
| Low neighbourhood SES | ref | ref | ref |
| Middle SES | 0.73 | 0.77 | 0.32 |
| High SES | 0.71 | 0.13 | **0.02** |
|  |  |  |  |
| Male | ref | ref | ref |
| Female | 0.22 | 0.93 | 0.07 |
|  |  |  |  |
| 18 – 35 years old | ref | ref | ref |
| 36 – 49 years old | 0.16 | 0.42 | **<0.001** |
| 50 – 65 years old | 0.37 | 0.44 | **<0.001** |
